# Development of a noninvasive murine model of hyperthermic intraperitoneal chemotherapy (HIPEC) treatment for ovarian cancer

**DOI:** 10.21203/rs.3.rs-10080092/v1

**Published:** 2026-07-07

**Authors:** Zahraa Alali, Olivia G. Patterson, Sam Lee Sangmyung, Max P. Horowitz, Danielle Chau, Rashmi Bharti, Jing Hao, Peng Qi, Emily E. Rhoades, Robert L. DeBernardo, Jennifer S. Yu, Ofer Reizes

**Affiliations:** Cleveland Clinic Research, Cleveland Clinic; Cleveland Clinic Research, Cleveland Clinic; Cleveland Clinic Research, Cleveland Clinic; Cleveland Clinic; Cleveland Clinic; Cleveland Clinic Research, Cleveland Clinic; Cleveland Clinic Research, Cleveland Clinic; Cleveland Clinic Research, Cleveland Clinic; Cleveland Clinic Research, Cleveland Clinic; Cleveland Clinic; Cleveland Clinic Research, Cleveland Clinic; Cleveland Clinic Research, Cleveland Clinic

## Abstract

Ovarian cancer (OC) is currently the leading cause of death from gynecological cancer, with 60% of cases ending in mortality. As an aggressive disease of the female reproductive system, OC in the advanced stage metastasizes to the peritoneum, and ascites is present in > 90% of patients with stage III–IV disease. This accumulation of fluid in the abdomen is associated with a 5% survival rate. Current therapeutic strategies exist for treating OC, yet tumors recur and become chemoresistance, indicating a critical need for improved therapeutics. Hyperthermic intraperitoneal chemotherapy (HIPEC) is a successful treatment given to select patients with advanced disease. HIPEC involves pumping heated chemotherapy throughout the peritoneal cavity at time of debulking surgery. HIPEC has shown success in treating advanced OC by extending overall survival by nearly 12 months, yet the mechanisms of benefit remain unknown. A rapid noninvasive animal model is lacking for the study of HIPEC. This poses a significant barrier for improving HIPEC protocols for patient benefit. We have developed the first noninvasive animal model by using external abdominal heat with chemotherapy. Results reveal significant attenuation of tumor growth, delayed tumor-associated ascites development, and extended survival after treatment with hyperthermic chemotherapy compared to normothermic cisplatin treatment. This method is rapidly allowing for multiple mouse treatments per procedure. Our findings show hyperthermic chemotherapy delays ascites onset and extend survival. This non-invasive model allows for implementation of mechanistic studies to investigate HIPEC survival benefit. Elucidating these mechanisms is essential to enhance HIPEC benefit and identifying strategies to define less invasive therapeutic mechanisms for the treatment of ovarian cancer.

## INTRODUCTION

Epithelial ovarian cancer (EOC) is the leading cause of gynecological cancer death in women with approximately 20,890 new cases diagnosed annually, 12,730 of which will succumb to the disease^1,2^. Despite standard of care neoadjuvant platinum-based chemotherapy and debulking surgery, more than 70% of patients experience disease recurrence within two years of initial diagnosis^3,4^. Most patients present at an advanced stage (stage III-IV) due to difficulty of early detection leading to poor prognosis. In the advanced stage, EOC metastasizes throughout the peritoneal cavity, with ascites developing in > 90% of patients. Ascites is an inflammatory response causing a buildup of fluid in the abdomen due to fluid leaking from organs and impaired lymphatic drainage^5,6^. This fluid accumulation further induces a tumor promoting environment and leads to chemoresistance in EOC patients^6^. EOC patients with ascites have a significantly poorer prognosis, with a 5-year survival of 5%, compared to 45% without ascites^7^.

Hyperthermic Intraperitoneal Chemotherapy (HIPEC) treatment for advanced EOC has emerged as a promising therapeutic approach. HIPEC treatment is given at the time of interval debulking surgery, after the bulk of the tumor and metastases are removed^4^. Heated chemotherapy, warmed to 42°C, is then pumped throughout the peritoneal cavity for a duration of 90-minutes at a constant temperature, in an attempt to target microscopic disease^4,8^. HIPEC has shown success in treating advanced EOC by extending overall survival by nearly 12 months. Advanced disease patients are selected based on development of peritoneal metastasis. Studies show that patients without a BRCA mutation have an increased benefit from HIPEC treatment compared to those with BRCA mutations, indicating that classification of gene mutations may be a key component in the study of HIPEC. HIPEC extends survival, yet a limitation of HIPEC treatment is the lack of understanding of the mechanistic benefit^9^. This poses a significant barrier for improving HIPEC protocols for patient benefit.

Given the lack of a rapid animal model of HIPEC, we sought to develop a non-invasive animal model to mimic clinical benefit of HIPEC. Currently available models are invasive with complex protocols that require the use of inflow/outflow catheters, lack reproducibility, and are performed in small cohorts of animals because of the time-consuming nature of survival surgery^10–19^. Currently available models are invasive with complex protocols that require the use of inflow/outflow catheters, lack reproducibility, and are performed in small cohorts of animals because of the time-consuming nature of the experimental setup^10–19^. Few models exist that allow for the safe and effective delivery of heated chemotherapy in multiple mice to analyze the mechanistic basis of HIPEC. Cisplatin is used clinically as a therapeutic in EOC patients and has shown success when used in murine models^20^.

We show here the development of a novel approach for the study of hyperthermia chemotherapy in a murine model of ovarian cancer. Addition of hyperthermia delivered externally via heated probes and a water pad with intraperitoneal injection of cisplatin leads to significantly decreased tumor growth compared to normothermic cisplatin. The benefit of hyperthermic chemotherapy leads to extended survival. Further, hyperthermia + cisplatin delayed tumor-associated ascites development, compared to normothermic cisplatin and saline controls. In summary, we developed the first reproducible noninvasive animal model of HIPEC for treatment of advanced ovarian cancer in a platform amendable to genetic, immunologic, and pharmacologic manipulation.

## MATERIALS AND METHODS

### Chemicals and Reagents

Cell culture medias (RPMI Cat. # 10–500; DMEM Cat. # 11–500) and Trypsin (T/E 1X) (Cat. #521 − 500) were purchased from the Lerner Research Institute Media Core at the Cleveland Clinic. FBS was purchased from Atlas Biologicals (Cat. #F-0500-D). Cisplatin (50 mg solution) (Cat #63323-0103-51) and Isoflurane (250 mL) (Cat. #66794-0017-25) were purchased from the Cleveland Clinic Pharmacy. D-luciferin (Goldbio Cat. #LUCK-5G), DMSO (Millipore-Sigma Cat. #D2650), MEM α (Gibco Cat. #12571048), Insulin-Transferrin-Selenium (ITS) (Gibco Cat. #41400045), Penicillin-Streptomycin (Gibco Cat. #15140122), epidermal growth factor (EGF; Sigma-Aldrich, Cat. #E4127), and Worthington Papain Dissociation Kit (Worthington Cat. #LK003153) were used for cell culture, imaging, freezing, and tissue dissociation.

### Murine Ovarian Cancer Cell Lines and Culture Conditions

Initial development of this murine model of HIPEC utilized ID8-luc cells. Murine derived epithelial ovarian cancer cells expressing luciferase (ID8-luc) were generated in the lab as previously described^21,22^. ID8-luc cells were cultured in Dulbecco’s Modified Eagle Medium (DMEM) containing 5% heat inactivated fetal bovine serum (FBS). For proof of concept, an additional cell line (IE9 cells) was utilized. IE9 cells are a derivative of ID8-luc cells which replicate human ovarian cancer in mice including the development of ascites in the advance stage of disease^23^. IE9 cells were cultured in Roswell Park Memorial Institute (RPMI) media containing 10% FBS. IE9 cells were provided by University of Chicago and were transduced via lentivirus to express luciferase, following an established protocol^24^. The BRCA1-mutated triple-knockout (TKO; *p53*^*−/−*^*Brca1*^*−/−*^*Pten*^*−/−*^) cells are derived from the murine fallopian tube epithelium and were gifted by Dr. Ronny Drapkin (University of Pennsylvania, Philadelphia, PA). TKO cells were cultured in MEM α supplemented with 10% FBS, 0.1% Insulin-Transferrin-Selenium (ITS) (Gibco #41400045), 2 ng/mL epidermal growth factor (EGF), and 1% Penicillin-Streptomycin (Gibco #15140122). All cells were grown under standard conditions in a humidified incubator at 5% CO_2_. At approximately 70–80% confluency, media was removed from cell culture plates, cell plates were washed with phosphate buffered saline (PBS) and treated with trypsin for detachment. ID8-luc, IE9, and TKO cell pellets were resuspended in PBS for injection into mice.

### Intraperitoneal Injection of ovarian cancer cells

6–8-week-old C57BL/6J female mice were purchased from Jackson Laboratories (Bar Harbor, ME, RRID IMSR_JAX:000664) and NSG mice were purchased from Cleveland Clinic Biological Resources Unit (BRU) in-house breeding. Mice were intraperitoneally (IP) injected with 5×10^6^ ID8-luc cells prepared in 300 μL FBS-free DMEM media, 3×10^6^ IE9 cells prepared in 200 μL PBS, or 3×10^6^ TKO cells prepared in 200 μL PBS using one 25-guage 5/8” needle syringe per mouse for injection. Mice were housed in standard housing in accordance with Cleveland Clinic Lerner Research Institute Biological Resources Unit protocols. All mouse procedures and treatments were approved by the Cleveland Clinic Lerner Research Institute Institutional Animal Care and Use Committee (IACUC) under protocol numbers 00002619, “Hyperthermic Intraperitoneal Chemotherapy (HIPEC) for sensitizing gynecologic cancers to adjuvant therapy,” and 00003211, “Investigating Mechanisms of Hyperthermic Intraperitoneal Chemotherapy (HIPEC) Sensitization of Ovarian Cancer to Adjuvant Therapy.”

#### In Vivo Imaging

Two weeks after cell injection, bioluminescence imaging of mice was performed to confirm tumor engraftment. Mice were intraperitoneally injected with 100 μL D-luciferin (Goldbio 150 mg/kg in 67 mL PBS) and anesthetized with inhaled isoflurane via isoflurane chamber prior to imaging. Mice were placed in the supine position in the IVIS Spectrum (PerkinElmer) system to obtain bioluminescence images. Nose cones providing isoflurane anesthesia are within the IVIS Imager to ensure mice are kept under constant anesthesia. Analysis of images was done using Living Image Software, and the intensity of the signal (total flux) was recorded. Imaging was repeated weekly until study endpoint. For quantification of IVIS imaging, the total photon flux of each time point, calculated by Living Image Software, was subtracted from the Day 0 pre-treatment measurement to evaluate the change.

### Hyperthermia Application and Cisplatin Administration

Two hyperthermia systems were evaluated and used during model development: the Pyrexar BSD500 Microwave Hyperthermia System and the ThermoTek T257P temperature-controlled water circulation system. Initial optimization and ID8-Luc experiments were performed using the BSD500 system in combination with a heated water pad, whereas subsequent experiments were performed using the ThermoTek T257P system with a heated water pad as the sole source of hyperthermia.

For experiments performed with the ThermoTek system, hyperthermia was administered using the ThermoTek T257P system connected to a heated water pad. Mice were anesthetized via an isoflurane chamber connected to a portable isoflurane anesthesia machine. To apply a heat treatment to the abdomen and pelvis, mice were placed in the prone position on top of a heated water pad set to a constant circulating 42°C. During treatment, the circulating water temperature was maintained and displayed by the ThermoTek T257P system at the programmed set point of 42°C. Mouse abdominal or core temperature was not continuously measured during ThermoTek-based treatments. The heated water pad provided reproducible heating without observable distress or internal burns and was used as the sole hyperthermia treatment in subsequent experiments. The circulating water temperature was monitored using the ThermoTek T257P display and maintained at a set point of 42°C throughout treatment. Mouse core and abdominal surface temperatures were not continuously measured during these experiments. Mice were kept under constant isoflurane anesthesia for the duration of a 20-minute heat treatment via a nose cone connected to the portable isoflurane anesthesia machine. The anesthesia machine remains connected to a wall-mounted oxygen supply with the oxygen flow meter set to 2–3L/min. At time of hyperthermia treatment, 100 μL cisplatin or saline was administered via IP injection. Mice were monitored for signs of distress or hyperventilation. The first dose of IP cisplatin was administered at the time of hyperthermia treatment, with additional IP cisplatin or saline given once weekly until study endpoint. For all studies with a two-week endpoint, two total doses of cisplatin or saline were administered, once at time of initial treatment and once the week following treatment. Hyperthermia treatment occurred once, after tumor engraftment was confirmed in mice.

### Control Mice Treatment Conditions

Two groups of control mice were utilized for the studies: V37 vehicle control and C37 cisplatin only control. V37 mice received a 100 μL IP saline injection and were kept at normothermic temperature. C37 mice received a 100 μL IP cisplatin injection and kept at normothermic temperature. V37 and C37 mice were kept at normothermic temperature for duration of the study. At time of hyperthermia treatment and initial injection of cisplatin or saline for treatment mice, all control mice were also placed under constant isoflurane anesthesia for consistency, however, both V37 and C37 mice were not placed on the hot water pad, but rather kept at normothermic temperature inside the isoflurane chamber for 20-minutes while the treatment mice were receiving the hyperthermic treatment on the hot water pad. Control mice were given initial dose of saline (V37) or cisplatin (C37) while under anesthesia at time of treatment mice receiving hyperthermia. For all studies with a two-week endpoint, two total doses of saline or cisplatin were administered, once at time of initial treatment and once the week following treatment.

### Mouse Necropsy and Sample Collection

At endpoint, mice were euthanized via CO_2_ asphyxiation and secondary cervical dislocation. Necropsy was performed with sterile surgical instruments and all samples collected for analysis. Mice were pinned in the supine position and sprayed with 70% ethanol to clean the external surface and moisten the fur. Ascites fluid was collected via 1 mL syringe with a 25-guage needle inserted gently into the peritoneal space prior to fully exposing the peritoneal cavity. Ascites fluid volume was collected and volume quantified. An incision using dissection scissors was made along the midline, exposing the abdominal organs and thoracic cavity. Blood was collected directly via cardiac puncture using a 25-guage needle attached to 1 mL syringe. Blood was placed in a 1.5 mL centrifuge tube and centrifuged at 4°C for serum collection. Tumors were harvested, weighed, and dissociated into a single cell suspension using a papain digestion protocol. Ascites cell pellet and tumor single cell suspensions were stored in 1 mL freeze media (90% FBS 10% dimethyl sulfoxide (DMSO)). All cryovials were placed in Mr. Frosty^™^ Freezing Containers (Thomas Scientific Cat #5100–0001) overnight for optimal slow cell preservation before storage in the − 80°C freezer.

### Statistical Analysis

Data were analyzed and graphed with GraphPad Prism 9.1 using Two-Way ANOVA and Tukey’s post hoc analysis. Survival curve was built using GraphPad Prism 9.1 using the Kaplan-Meier survival analysis. Survival statistical analysis was analyzed via log-rank test. For all statistical analyses p < 0.05 was considered statistically significant.

## RESULTS

### Optimization of Hyperthermia Treatment in Immunocompetent C57BL/6J Mice

We evaluated two approaches for delivering external abdominal hyperthermia: the Pyrexar BSD500 Microwave Hyperthermia System used in combination with a heated water pad, and the ThermoTek T257P temperature-controlled water circulation system used with a heated water pad alone. The BSD500-based approach was evaluated first during optimization and was used for the initial ID8-Luc studies, whereas the ThermoTek system was adopted for subsequent experiments after the microwave antenna was discontinued.

The BSD500 Microwave Hyperthermia System is equipped with eight channels which may be adjusted to a desired temperature (40°C) and wattage to provide radiant energy. This radiant energy allows for heat administration applied to the area of interest (abdominopelvic region) (Supplemental Fig. 2).

To evaluate the safety and the optimal time and temperature of the procedure, tumor-free C57BL/6J mice were subjected to several conditions in three separate trials: (1) 42°C heat treatment for 90-minutes, (2) 42°C heat treatment for 40 minutes, (3) 42°C heat treatment for 20 minutes. Trial 1 included the use of a microwave antenna (915 MHz) affixed under the abdomen of each mouse and a hot water pad set to a continuous 42°C. Trial 1 was stopped after 50 minutes due to mouse mortality and signs of respiratory distress in remaining mice. Trial 2 included the use of a microwave antenna (915 MHz) affixed under the abdomen of each mouse and a hot water pad set to a continuous 42°C and was stopped after 30 minutes when all mice showed signs of respiratory distress. Necropsy of these mice showed internal burning resulting from the microwave antenna. Adjustments were made in Trial 3 to account for poor outcomes: the temperature of the microwave antenna was reduced to 40°C and continued for 20 minutes, while the heated water pad remained set to 42°C. The decrease in temperature of the microwave antenna to 40°C initially eliminated the issue of burned tissue and was tolerated well by mice. These changes resulted in a safe induction of heat without complication or mortality. A thermistor probe to monitor real-time temperature was placed adjacent to the microwave antenna for each mouse to ensure the abdomen of each mouse reaches 42°C. However, we found the thermistor probe does not provide the ability to record a continuous 42°C temperature for the 20-minute heat treatment, rather the thermistor probe only allows for in-treatment temperature measurements.

**Figure 1:**
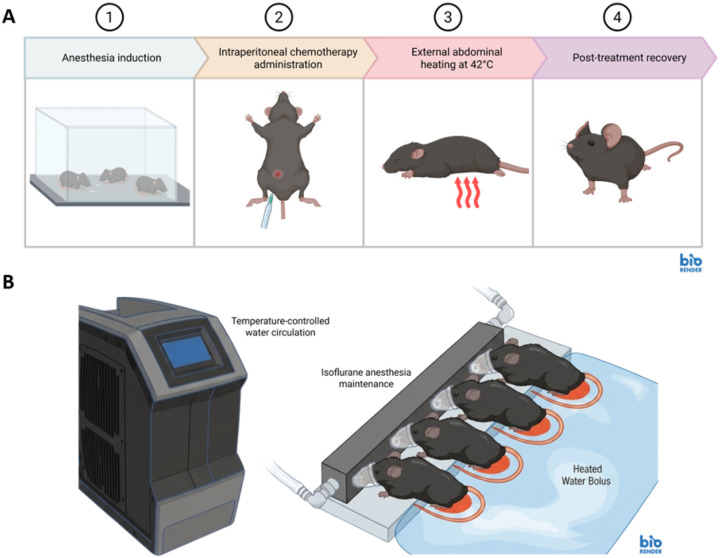
Murine hyperthermia-assisted chemotherapy workflow and treatment platform. **(A)** Experimental workflow for hyperthermia-assisted chemotherapy treatment. Mice are first induced under inhaled isoflurane anesthesia, followed by intraperitoneal chemotherapy administration. Mice then undergo external abdominal heating at 42°C and are subsequently monitored during post-treatment recovery. **(B)** Schematic of the hyperthermia treatment platform. During treatment, mice are maintained under isoflurane anesthesia through a nose cone manifold and positioned in the prone position on a flexible heated water bolus. Temperature-controlled water circulation is provided by a ThermoTek T257P water circulator to maintain external abdominal heating during treatment.

Although the modified BSD500 protocol was initially tolerated, a further complexity is that we discovered the microwave antenna led to intermittent internal burning. We stopped the use of the microwave antenna as the heating source and relied on the 42°C heated water pad to provide external heat treatment. Consistent and reproducible results were observed with 42°C hot water pad alone for delivery of heat treatment. Based on these optimization studies, the initial ID8-Luc experiments were completed using the optimized BSD500-based protocol. For subsequent model development and treatment experiments, the microwave antenna was discontinued, and hyperthermia was administered using the ThermoTek T257P system with a heated water pad set to a continuous circulating temperature of 42°C (Fig. 1A). The heated water pad alone provided consistent and reproducible heat delivery and allowed simultaneous treatment of up to 10 mice (Fig. 1B).

### Impact of Hyperthermia Treatment in the Presence and Absence of Cisplatin in Murine EOC

**Figure 2:**
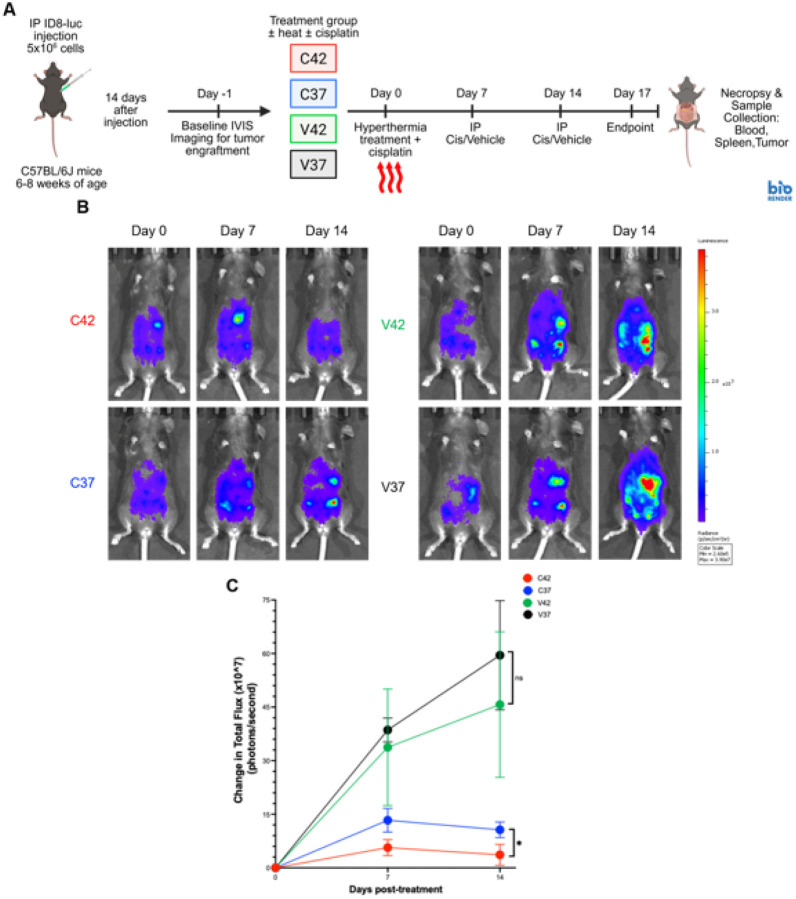
Treatment of EOC mouse model with presence and absence of hyperthermic cisplatin. **(A)** Treatment timeline of EOC tumor bearing mice treated with hyperthermic cisplatin. C42: hyperthermia + cisplatin; C37: cisplatin alone; V42: hyperthermia alone; V37: saline vehicle alone. *In Vivo* Imaging Systems (IVIS); intraperitoneal (IP) injection. **(B)** Representative bioluminescence images of each group from pre-treatment to 7 and 14 days after treatment. **(C)** Change in bioluminescence signal (total flux) at Day 0 (pre-treatment) and at days 7 and 14 post-treatment. C42 and C37 mice showed significant attenuation in tumor burden via decreased total flux signal 14 days after treatment. Graphed data mean ± SEM; * P < 0.05. Graphed data mean ± SEM; *, p < 0.05. **Mouse cohorts:** C42: mice treated with 5 mg/kg cisplatin and 42°C hyperthermia (n=12); C37: mice treated with 5 mg/kg cisplatin and kept at normothermic temperature (n=12); V42: mice treated with saline and 42°C hyperthermia (n=8); V37: mice treated with saline and kept at normothermic temperature (n=12). Two technical replicates. Two-Way ANOVA mixed effects analysis used for statistical analyses.

C57BL/6J mice were intraperitoneally injected with ID8-luc cells prior to treatment. A schematic representation of the treatment timeline is shown in Fig. 2A. Two-weeks after injection, IVIS imaging was used to confirm tumor engraftment, which showed mice had developed abdominopelvic metastases (Fig. 2B). The observed peritoneal dissemination is representative of findings in human ovarian cancer^25^. Mice were randomized into treatment groups: hyperthermia + cisplatin (C42), cisplatin alone (C37), hyperthermia + saline (V42), and saline alone (V37). At time of treatment, mice received an IP injection of either 200 μL cisplatin or saline (vehicle). C42 and V42 mice were subsequently anesthetized via isoflurane and immobilized on the hyperthermia device via nose cones providing constant isoflurane anesthesia. ID8-Luc-bearing mice received hyperthermia using the optimized BSD500 protocol consisting of a 40°C superficial microwave antenna combined with a 42°C heated water pad for 20 minutes. The abdominopelvic regions were heated to 42°C, while C37 and V37 mice were maintained at normothermic temperature (37°) within the anesthesia chamber. An underlying water circulation system (heated water pad) set to 42°C was used for the even distribution of heat treatment and to ensure stabilization of body temperature. Superficial temperatures of the animals were monitored in real time using thermistor probes to ensure stable body temperature was maintained for the duration of hyperthermic treatment. No animal deaths were recorded during the procedure and mice tolerated the heat with no observable adverse effects. IVIS analysis was completed at Day 7 and Day 14 after treatment (Fig. 2B) and analyzed for bioluminescence signal changes between treatment groups (Fig. 2C). Change in bioluminescence (total flux) showed a significant reduction in tumor burden among the C42 and C37 groups, compared to saline controls. Hyperthermia + cisplatin mice had a significant reduction in tumor growth compared to normothermic cisplatin treatment. Results indicate the combination of hyperthermia and cisplatin treatment in mice significantly reduces tumor growth, compared to cisplatin alone and heat alone.

**Figure 3:**
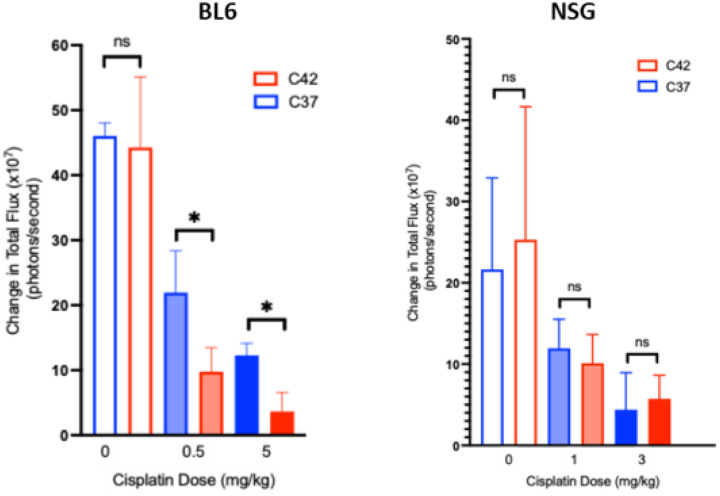
The immune system is essential in HIPEC benefit. Change in bioluminescence signal (total flux) two weeks after treatment compared to pre-treatment IVIS signal in C75Bl/6J and NSG mice. Hyperthermic chemotherapy effect is lost in absence of immune system, as seen by dose response study done in NSG mice.

C57BL/6J mice were IP injected with ID8-luc cells and IVIS imaging confirmed tumor engraftment two weeks after injection, prior to any treatment. Mice were given treatments of cisplatin alone or hyperthermia + cisplatin, with varying doses of cisplatin in a dose dependent study. IVIS imaging via bioluminescence was quantified and change in total flux was recorded (Fig. 3). Mice treated with hyperthermia exhibited a significant reduction in tumor growth compared to the normothermic controls when dosed with cisplatin. We observed a dose dependent decrease in tumor growth in response to cisplatin treatment (0.5 and 5 mg/kg) that was further decreased by hyperthermia treatment. Hyperthermic chemotherapy effect is lost in absence of immune system, as seen by dose response study done in NSG mice. Our results suggest a synergy between hyperthermia and cisplatin, as the effect of tumor growth attenuation is greater with application of hyperthermia.

### A combination of hyperthermia + cisplatin treatment delays development of ascites

**Figure 4:**
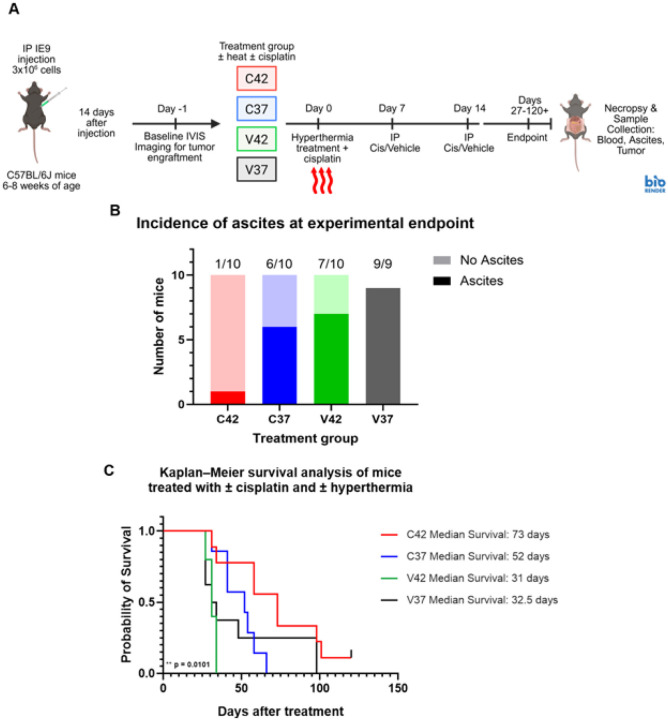
Hyperthermic cisplatin treatment delays ascites onset and extends survival in ovarian cancer bearing mice. **(A)** Schematic representation of treatment timeline. Mice were injected and treated as previously described. Survival study was built by following mice until obvious abdominal distension was observed (ascites), at which time necropsy occurred. **(B)** Mice were injected with IE9 ovarian cancer cells and treated as previously described in Fig. 1A. At experimental endpoint, C42 markedly reduced ascites incidence. Ascites incidence differed significantly among treatment groups by Fisher–Freeman–Halton exact test for a 4 × 2 contingency table (**p = 0.0004**). **(C)** Mice treated with hyperthermia + cisplatin had extended survival. Endpoint was defined as ascites development via abdominal distension, at which time mice were euthanized, and death date was recorded as days after treatment and recorded in survival curve. Log-rank test used for statistical analysis. **, p = 0.0101. **Mouse cohorts:** C42: mice treated with 1 mg/kg cisplatin and 20 minutes of 42°C hyperthermia; C37: mice treated with 1 mg/kg cisplatin and kept at normothermic temperature; V42: mice treated with saline and 20 minutes of 40°C hyperthermia; V37: mice treated with saline and kept at normothermic temperature. (C) C42 n=10, C37 n=10, V42 n=7, V37 n=8.

We assessed the impact of hyperthermia + cisplatin on a mouse model that develops ascites. The IE9 cell line, a variant of ID8, has been shown to develop ascites in mice^21^. Mice were injected with IE9 cells and monitored for 8 weeks to allow sufficient tumor establishment and ascites development before initiating hyperthermia plus cisplatin treatment. Mice were randomized into treatment groups: abdominal hyperthermia + cisplatin (C42), cisplatin alone (C37), hyperthermia + saline (V42), and saline alone (V37). Mice received either 1 mg/kg cisplatin or saline at time of normothermia or hyperthermia treatment and once weekly until endpoint (Fig. 4A). Mice were monitored for two weeks following treatment, for a total of 10-weeks after injection to complete the study. At the time of necropsy, ascites fluid was collected, and the volume was recorded for each mouse. C42 mice showed no signs of ascites development, compared to all other treatment groups from which ascites was collected. Hyperthermia plus cisplatin markedly reduced ascites incidence at endpoint. Ascites was observed in only 1/10 C42 mice, compared with 6/10 C37, 7/10 V42, and 9/9 V37 mice (Fig. 4B). Ascites incidence differed significantly across treatment groups (p = 0.0004) using a Fisher–Freeman–Halton exact test for a 4 × 2 contingency table. Planned pairwise comparisons using two-sided Fisher’s exact tests showed reduced ascites incidence in C42 mice compared with V42 and V37 groups, while the comparison between C42 and C37 showed a strong trend but did not reach significance by two-sided testing. These findings support that combined hyperthermia and cisplatin treatment suppresses ascites development in IE9 ovarian cancer-bearing mice..

### Hyperthermia + cisplatin treatment in C57BL/6J IE9 tumor bearing mice extends survival

The lack of ascites development in C42 mice two weeks after treatment with hyperthermia + cisplatin led us to assess overall survival. Tumor bearing C57BL/6J mice were injected intraperitoneally with IE9 cells and monitored for 8 weeks to allow sufficient tumor establishment and ascites development before treatment. Mice then received hyperthermic or normothermic treatment with and without weekly doses of IP cisplatin (1 mg/kg) injection and monitored until endpoint. Onset of ascites development based on abdominal distension was the endpoint criteria. The endpoint date for each mouse was used to analyze and generate a survival curve (Fig. 4C). Survival analysis reveals hyperthermia + cisplatin significantly extends mouse survival with a median survival to 73 days, compared to 52 days after treatment with cisplatin alone. Hyperthermic treatment alone did not impact mouse survival, suggesting the combination of hyperthermia and cisplatin is required for survival benefit.

### BRCA-mutated tumor bearing mice exhibit no benefit from treatment with hyperthermia + cisplatin

**Figure 5:**
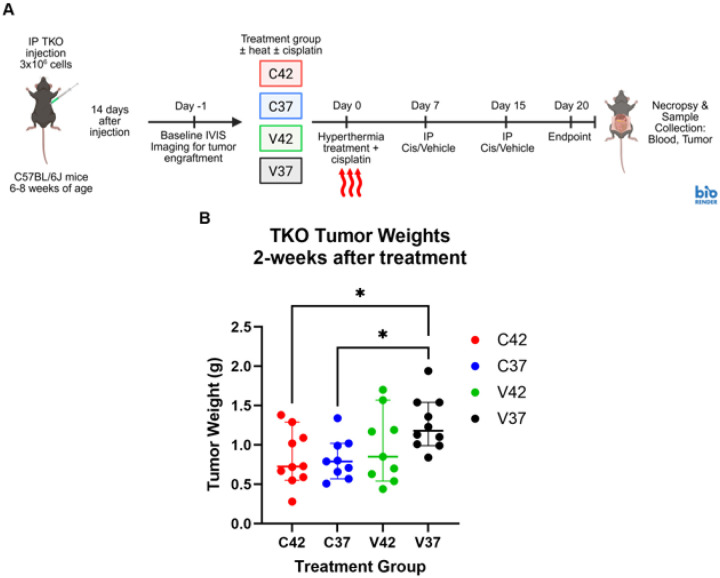
BRCA-mutated ovarian cancer bearing mice lose the benefit of hyperthermia + cisplatin treatment. **(A)** Schematic representation of treatment timeline. Mice were injected and treated as previously described. **(B)** No difference in tumor growth attenuation was seen between hyperthermia plus cisplatin and cisplatin alone, consistent with clinical findings that BRCA-mutated tumors do not respond well to HIPEC. **Mouse cohorts:** C42 (n=10): mice treated with 1 mg/kg cisplatin and 20 minutes of 42°C hyperthermia; C37 (n=9): mice treated with 1 mg/kg cisplatin and kept at normothermic temperature; V42 (n=9): mice treated with saline and 20 minutes of 40°C hyperthermia; V37 (n=10): mice treated with saline and kept at normothermic temperature. Two-Way ANOVA used for statistical analyses.

Clinical data suggests ovarian cancer patients with a BRCA mutation lose the benefit from HIPEC treatment^26^. A murine cell line containing a BRCA mutation was used to test the efficacy in hyperthermic chemotherapy function^26^. The TKO cell line is a murine derived BRCA mutated ovarian cancer cell line. We used the TKO cell line in our hyperthermia model (Fig. 5A). Two weeks after injection with TKO cells, C57BL/6J mice received hyperthermic or normothermic treatment with and without cisplatin (1 mg/kg). Mice received a 20-minute abdominal heat treatment and monitored for signs of distress. Hyperthermia + cisplatin and cisplatin alone mice received two additional dose of 1 mg/kg cisplatin IP two week following treatment, with endpoint being 20 days after treatment. At study endpoint, mice were euthanized before collection of blood and tumor. Tumor weights were recorded and showed no significant difference between hyperthermia plus cisplatin and cisplatin alone (Fig. 5B). The lack of difference in tumor weight between groups is similar to clinical observations that BRCA-mutated ovarian cancer patients do not benefit from HIPEC^26^.

## DISCUSSION

We developed a noninvasive method of hyperthermia delivery that is reproducible in large cohorts and provides a platform to treat multiple mice with hyperthermia. We utilized a simple water bath format to expose the mice hyperthermia. The format allows for treatment of 5 mice at a time under multiple conditions to elucidate mechanisms of hyperthermia and cisplatin on ovarian cancer. These studies indicate that hyperthermia + cisplatin treatment in EOC bearing mice limits tumor growth, delays ascites onset, and extends mouse survival.

### The development of a noninvasive murine model of HIPEC

Our studies focused on a rapid, noninvasive model of HIPEC. We found that by using a combination of external abdominal heat and cisplatin, tumor growth was limited compared to treatments with hyperthermia alone and normothermic cisplatin. Current murine models of HIPEC lack the ability to provide treatment to a large cohort of mice and fail to show long-term survival after treatment. Our treatment model showed extension of mouse survival when mice were treated with hyperthermic cisplatin, which mimics clinical HIPEC results of extending overall survival^9^.

### Noninvasive murine hyperthermic chemotherapy model mimics clinical HIPEC

We tested the reproducibility of our newly developed hyperthermia chemotherapy model by using multiple murine ovarian cancer cell lines. The IE9 cell line mimics clinical disease via development of ascites in the advanced stage. Treatment of IE9 bearing mice revealed hyperthermia + cisplatin treated mice did not develop ascites two weeks after treatment compared to control groups. Nearly all tumor bearing mice treated with normothermic cisplatin, hyperthermic saline, and normothermic saline mice contained ascites at necropsy. The lack of ascites in hyperthermic cisplatin treated tumor bearing mice led us to question if hyperthermia + cisplatin treatment eliminated ascites development altogether or delayed the onset of development.

Our survival study answered this question, showing a significant extension in survival when treated with hyperthermia + cisplatin. Considering ascites development in clinical EOC patients is accompanied by adverse effects, showing that hyperthermic cisplatin treatment may delay the onset of abdominal fluid buildup is a key component in beginning to understand the mechanisms of HIPEC benefit in extending patient survival. My goal was to show that this noninvasive model of hyperthermic cisplatin was sufficient to mimic clinical HIPEC. Clinical HIPEC has been shown to extend patient overall survival by nearly 12 months^9^. Our model shows an extension in survival of mice when using ascites as a basis for endpoint. Patients with a BRCA mutation have no added benefit from HIPEC treatment^26^. Our studies indicated that BRCA-mutated murine cells in mice do not benefit from hyperthermia plus cisplatin treatment. BRCA-mutated tumor bearing mice showed no difference in tumor growth attenuation after treatment with hyperthermia + cisplatin. Our results are consistent with clinical studies showing BRCA-mutated ovarian cancer patients lose HIPEC benefit^26^. Further understanding the mechanistic benefit of HIPEC would allow for advancement of treatment, potentially allowing for a broader range of qualifying patients to receive HIPEC, i.e. patients with a BRCA mutation.

### HIPEC benefit may stem from delaying ascites development

In BL6 mice bearing IE9 tumors, hyperthermic cisplatin treatment reduced ascites development at early post-treatment assessment and significantly delayed ascites onset over the course of the survival study.. Ascites development creates a chemo-resistant and tumor promoting environment, thus the hyperthermic cisplatin treatment delaying ascites onset may allow for the chemotherapeutics to target disease more efficiently, thus extending patient survival. Further analysis of the mechanism of ascites development after HIPEC is critical for understanding how treatment extends survival.

### Strengths and limitations of the hyperthermic cisplatin murine model of HIPEC

Clinical HIPEC treatment is completed at time of interval debulking surgery, therefore analysis of a decrease in tumor burden after treatment is not completed in human patients because their tumors are removed prior to HIPEC treatment. A limitation of our murine model is that tumor burden is not removed prior to the hyperthermic cisplatin treatment. This provides an advantage because it allows for the analysis of tumor growth changes throughout the study; however, the presence of tumor at time of hyperthermic cisplatin treatment is a limitation as it is not equivalent to what is seen in clinical patients at time of HIPEC treatment. Additionally, the presence of tumor at time of treatment may not allow for the hyperthermic cisplatin treatment to provide full benefit due to difficulty reaching bulk tumors.

Our murine model lacks the ability to confirm internal abdominal temperature is reaching the desired 42°C during hyperthermic treatment. In the future, the addition of a rectal thermometer to measure mouse body temperature during or immediately after hyperthermic treatment would be beneficial to confirm the external heat is sufficient in providing internal hyperthermia.

Our murine model utilizes an external heat treatment, which does not exactly replicate the invasive nature of clinical HIPEC; however, our goal was to develop a noninvasive model that mimics clinical results without using an invasive perfusion system. Clinical HIPEC circulates heated chemotherapy throughout the peritoneal cavity. The external heat treatment we used may pose a limitation with a lack of circulating hyperthermic cisplatin. The chemotherapeutic agent (cisplatin) in clinical HIPEC is heated to 42°C prior to introduction into the peritoneal cavity of clinical patients. Our model may pose a limitation in that the cisplatin itself is not heated to 42°C, but rather is injected into the peritoneal cavity immediately before the external heat is applied. It is unclear if after injection the cisplatin reaches the desired temperature while within the mouse during heat treatment. The future use of this model may consider heating the cisplatin to 42°C prior to injection into the mouse to more closely mimic clinical HIPEC procedure.

A strength of our model is that multiple mice can be treated at one time, making the treatment model more efficient. Mice tolerate the treatment well with little to no respiratory distress. Survival of mice is also reported after hyperthermic cisplatin treatment, which is a novel finding in the study of hyperthermic chemotherapy in mice. Our treatment model is noninvasive, extends survival may overcome some of the complications typically seen from an invasive HIPEC treatment in mice.

## CONCLUSIONS

We provide evidence for a simple hyperthermic chemotherapy murine model for mimicking clinical HIPEC. This model is rapid and allows for the analysis of multiple manipulations. Our studies show that hyperthermic cisplatin delays the onset of ascites, which requires more analysis to determine the mechanism behind the development of fluid buildup after cancer treatment. Elucidating the mechanistic benefit of HIPEC is crucial in improving therapeutics to make HIPEC treatment more accessible, less invasive, and ultimately further extend patient survival. We have successfully developed a noninvasive hyperthermic chemotherapy murine model showing extension of mouse survival, a delay in ascites development, and attenuation of tumor growth which can be used in future studies to uncover the mechanisms of HIPEC benefit.

## Data Availability

The datasets used and/or analyzed during the current study are available from the corresponding author on reasonable request.

## Abbreviations

All abbreviations were defined the first time they appeared in each article section.

BRU: Biological Resources Unit
DMEM: Dulbecco’s Modified Eagle Medium
DMSO: dimethyl sulfoxide
EGF: epidermal growth factor
EOC: epithelial ovarian cancer
FBS: fetal bovine serum
HIPEC: Hyperthermic Intraperitoneal Chemotherapy
IACUC: Institutional Animal Care and Use Committee
IP: intraperitoneal
ITS: insulin-transferrin-selenium
IVIS: *in vivo* imaging systems
OS: overall survival
PBS: phosphate buffered saline
RPMI: Roswell Park Memorial Institute
